# Detection of bone erosions in rheumatoid arthritis wrist joints with magnetic resonance imaging, computed tomography and radiography

**DOI:** 10.1186/ar2378

**Published:** 2008-02-28

**Authors:** Uffe Møller Døhn, Bo J Ejbjerg, Maria Hasselquist, Eva Narvestad, Jakob Møller, Henrik S Thomsen, Mikkel Østergaard

**Affiliations:** 1 Department of Rheumatology, Copenhagen University Hospital Hvidovre, Kettegaard Allé 30, 2650 Hvidovre, Denmark; 2 Department of Diagnostic Radiology, Copenhagen University Hospital Herlev, Herlev Ringvej 75, 2630 Herlev, Denmark; 3 Department of Radiology, Copenhagen University Hospital Rigshospitalet, Blegdamsvej 1, 2100 Copenhagen, Denmark; 4 Department of Rheumatology, Copenhagen University Hospital Herlev, Herlev Ringvej 75, 2630 Herlev, Denmark

## Abstract

**Background:**

The objectives of the present study were, with multidetector computed tomography (CT) as the reference method, to determine the performance of magnetic resonance imaging (MRI) and radiography for the detection of bone erosions in rheumatoid arthritis wrist bones, and to test whether measuring volumes of erosions on CT and MRI is reproducible and correlated to semiquantitative assessments (scores) of erosions on CT, MRI and radiography.

**Methods:**

Seventeen patients with rheumatoid arthritis and four healthy control individuals underwent CT, MRI and radiography of one wrist, performed on the same day. CT was performed on a Philips Mx8000IDT unit (voxel size 0.4 mm × 0.4 mm × 1 mm) and MRI was performed on a Philips Panorama 0.6T unit (voxel size 0.4 mm × 0.4 mm × 0.4 mm). Images were evaluated separately for erosions in all wrist bones and were scored according to the principles of the Outcome Measures in Rheumatology Rheumatoid Arthritis MRI Scoring System (CT and MRI) and the Sharp/van der Heijde (radiographs) scoring methods. Measurements of erosion volumes of all erosions were performed twice with a 1-week interval.

**Results:**

With CT as the reference method, the overall sensitivity, specificity and accuracy (concordance) of MRI for detecting erosions were 61%, 93% and 77%, respectively, while the respective values were 24%, 99% and 63% for radiography. The intramodality agreements when measuring erosion volumes were high for both CT and MRI (Spearman correlation coefficients 0.92 and 0.90 (both *P *< 0.01), respectively). Correlations between volumes and scores of individual erosions were 0.96 for CT and 0.99 for MRI, while they were 0.83 (CT) and 0.80 (MRI) for persons' total erosion volume and total score (all *P *< 0.01).

**Conclusion:**

With CT as the reference method, MRI showed moderate sensitivity and good specificity and accuracy for detection of erosions in rheumatoid arthritis and healthy wrist bones, while radiography showed very low sensitivity. The tested volumetric method was highly reproducible and correlated to scores of erosions.

## Introduction

Radiography, traditionally considered the golden standard for assessing structural joint damage in patients with rheumatoid arthritis (RA), is used routinely for diagnosing and monitoring RA patients, and is used as an endpoint in clinical trials [1,2]. In early undifferentiated arthritis, the presence of bone erosions is a risk factor for developing persisting arthritis [3], and the presence of erosions when diagnosing RA is related to a poor long-term functional and radiographic outcome [4-8]. For these reasons, detection of erosions as early as possible is desirable. Radiography does not visualise the earliest stages of erosive changes in RA, however, and other imaging modalities have emerged as methods for more sensitive detection of early bone erosions [9-12].

Magnetic resonance imaging (MRI) has been demonstrated to be more sensitive than radiography in detecting erosive bone changes in RA, especially the subtle changes that occur in early disease [9-11,13,14]. The Outcome Measures in Rheumatology (OMERACT) Rheumatoid Arthritis MRI Scoring System (RAMRIS) has been developed [15,16] with data from iterative multicenter studies [15,17,18]. The OMERACT RAMRIS is a semiquantitative scoring system for assessing synovitis, bone erosions and bone edema on MRI in RA hands and wrists. Studies on volumetric quantification of bone erosion volumes with MRI have previously shown it is a reliable and feasible method [19-21], and it could possibly be beneficial in documenting progression or regression of structural joint damage in longitudinal studies.

Multidetector computed tomography (CT) is a tomographic radiographic imaging method offering isotropic high-resolution and three-dimensional visualisation of calcified tissue. CT seems to be even more sensitive than MRI for detection of bone erosions, and can be considered a standard reference for detection of bone erosions in RA [12,22,23].

An objective of the present cross-sectional methodological study was, with CT as the reference method, to investigate the sensitivity, specificity and accuracy (concordance) of MRI and radiography for detection of bone erosions in RA wrist bones. A second objective was to determine the intramodality and intermodality agreement when measuring erosion volumes on CT and MRI in RA wrist bones, using a semiautomated computerised method. A third objective was to evaluate whether semiquantitative scoring methods for bone erosions (the OMERACT erosion score and the Sharp/van der Heijde radiographic erosion score) correlated with erosion volumes determined with CT and MRI.

## Patients and methods

### Patients and control individuals

Seventeen RA patients fulfilling the American College of Rheumatology 1987 criteria [24] – of which 14 were rheumatoid factor positive – and four healthy control individuals were included in the study. Fourteen patients were female and three were male (median age 51 years (range 33–78 years), median disease duration 7 years (range 4–22 years)), and three control individuals were female and one was male (median age 36 years (range 34–57 years)). All individuals underwent CT, MRI and radiography of one wrist joint on the same day. The study was approved by the local ethics committee, and written informed consent was obtained from all participants.

### Computed tomography

A Philips Mx8000 IDT multidetector unit (Philips Medical Systems, Cleveland, OH, USA) was used for all examinations (parameters: 90 kV, 100 mAs, pitch 0.4 mm, slice spacing 0.4 mm, overlap 50%). Patients were placed in a prone position with the arm stretched and the palm facing down. Images with a voxel size of 0.4 mm × 0.4 mm × 1.0 mm were obtained. Axial and coronal reconstructions with a slice thickness of 1.0 mm were created and used for image evaluation.

### Magnetic resonance imaging

A Philips Panorama 0.6 T unit (Philips Medical Systems, Helsinki, Finland) using a receive-only, three-channel, phased solenoid coil was used for all examinations. Patients were placed in a supine position with the hand alongside the body and the palm facing the body. Acquired images included a coronal T1-weighted three-dimensional fast field echo (repetition time 20 ms, echo time 8 ms, flip angle 25°, voxel size 0.4 mm × 0.4 mm × 0.4 mm, matrix 216 × 216, number of acquisitions 2, acquisition time 5.23 min). Images in the axial and coronal planes with a slice thickness of 0.4 mm were created by multiplanar reconstruction of the T1 three-dimensional fast field echo sequence, and these were used for image evaluation.

### Conventional radiography

Radiography was performed on a Philips Digital Diagnost unit (Philips Medical Systems, Hamburg, Germany) (resolution 0.3 mm). Posterior-anterior and semisupine projections were obtained and were printed on mammography films.

### Image evaluation

Images obtained with CT, MRI and radiography were evaluated for erosions by separate investigators, blinded to clinical and other imaging data, with large experience from previous imaging studies on RA. Erosions were marked on preformed scoring sheets, allowing exact positioning in all three planes, and an erosion score was assigned as described below.

Definitions of MRI erosions were as suggested by OMERACT RAMRIS; that is, a sharply marginated bone lesion, with correct juxtaarticular localisation and typical signal characteristics, visible in two planes with a cortical break seen in at least one plane [15]. MRI bone erosions were scored according to the OMERACT RAMRIS; that is, all wrist bones were assigned a score by the percentage of bone volume involved (score 0–10, by 10% volume increments) [15,25], leading to a total erosion score for one wrist ranging from 0 to 150.

Erosions on CT images were defined as a sharply demarcated area of focal bone loss seen in two planes, with a cortical break (loss of cortex) seen in at least one plane. CT bone erosions were scored according to the principles of the OMERACT RAMRIS method described above.

We applied the principles from the Sharp/van der Heijde scoring method in assessing radiographs, assigning an erosion score ranging from 0 to 5 to all wrist bones [26]. Briefly, individual erosions are given a score of 1 when discrete, a score of 2 if larger and a score of 3 when the erosion extends over the imaginary middle of the bone. If more than one erosion is present in a single bone, the sum of the scores (with a maximum of 5) of the individual erosions is calculated. With this modification of the scoring method, the total erosion score of one wrist ranges from 0 to 75.

### Erosion volume measurements

Owing to the severity of bone damage or ankylosis we excluded two patients from the analysis on erosion volume, leaving 19 patients and 285 bones for further analysis. The volumes of all erosions in the remaining 19 persons, detected by CT or MRI in the evaluation described above, were calculated using OsiriX medical imaging software (a free DICOM viewer for Apple computers that can be downloaded [27]). To calculate the erosion volume, erosions were manually outlined on coronal images, on all slices where visible. The outlining of erosion borders was done using an Intous3 A5 pen tablet system (Wacom Technology Corporation, Vancouver, WA, USA). The erosion volume is calculated by the software, according to the formula: Vol_ero _= Σ(Area_ero _x ST), where Vol_ero _is the erosion volume, Area_ero _the erosion area on one slice and ST is the slice thickness. All erosion volume measurements were performed by the same person (UMD) on two occasions with a 1-week interval between measuring on the same sets of images.

### Statistical analysis

The specificity, sensitivity and accuracy of MRI and radiography, with CT as the reference method, were calculated for bone erosions. To determine the reliability of erosion volume measurements, the absolute and relative differences, Spearman's correlation coefficients and the coefficient of variation of erosion volumes obtained with CT and MRI at the two readings (intramodality agreement) were calculated. Spearman's correlation coefficients were calculated between the OMERACT erosion scores and the erosion volumes of individual erosions and between the persons' total OMERACT erosion score and the persons' total erosion volumes (sum of the 15 evaluated joint areas). For erosions that were seen on both CT and MRI – that is, concordant erosions – the absolute and relative differences between CT and MRI erosion volumes (intermodality agreement) were calculated. Furthermore, intermodality agreements were assessed by calculation of Spearman's correlation coefficients and coefficients of variation. Correlation coefficients between the erosion volume, CT and MRI erosion scores and the radiographic erosion score were calculated. For calculation of intermodality agreement, the mean value of the volumes found at the two readings of CT respective to MRI was used. SPSS version 12.0 for Windows (SPSS Inc., Chicago, IL, USA) was used for statistical calculations.

## Results

In total, 315 wrist bones from 21 persons were assessed for erosions. A total of 166 erosions in 151 bones were detected with CT, while 119 erosions in 104 bones were detected on MRI, and 43 erosions in 38 bones were detected with radiography. With CT as the reference method for bone erosions, the overall sensitivity, specificity and accuracy of MRI were 61%, 93% and 77%, respectively. The corresponding values for radiography were 24%, 99% and 63%, respectively. Of the 119 MRI erosions, 92 (77%) could be confirmed with CT, whereas 36 (84%) of the 43 radiographic erosions were confirmed with CT. If considering only bones without radiographic erosions (*n *= 277), the overall sensitivity, specificity and accuracy of MRI were 59%, 93% and 79%, respectively. See Table 1 for further details.

**Table 1:** Sensitivities, specificities and accuracies for bone erosions of radiography and magnetic resonance imaging (MRI), with computed tomography (CT) as reference

	Bones with erosions (number of erosions)			Radiography			MRI			MRI values in bones without radiographic erosions (*n *= 277)		
	CT	Radiography	MRI	Sensitivity (%)	Specificity (%)	Accuracy (%)	Sensitivity (%)	Specificity (%)	Accuracy (%)	Sensitivity (%)	Specificity (%)	Accuracy (%)

Radius	10 (11)	2 (3)	6 (8)	20	100	62	60	100	81	50	100	79
Ulna	15 (15)	2 (2)	14 (15)	13	100	38	93	100	95	92	100	95
Scaphoid	11 (14)	3 (3)	8 (8)	27	100	62	64	90	76	50	90	72
Lunate	10 (11)	3 (3)	11 (14)	30	100	67	90	82	86	86	82	83
Triquetrum	14 (17)	5 (5)	13 (16)	36	100	57	86	86	86	100	86	94
Pisiforme	8 (8)	4 (5)	1 (1)	38	92	71	13	100	67	20	100	76
Trapezium	8 (8)	3 (5)	3 (3)	25	92	67	38	100	76	33	100	78
Trapezoid	8 (10)	2 (2)	9 (9)	25	100	71	86	85	86	83	85	84
Capitate	14 (14)	1 (1)	12 (16)	7	100	38	71	71	71	69	71	70
Hamate	9 (10)	3 (4)	7 (8)	33	100	71	56	83	71	50	85	71
Metacarpal base 1	8 (9)	3 (3)	5 (5)	38	100	76	63	100	86	40	100	83
Metacarpal base 2	16 (19)	1 (1)	9 (10)	6	100	29	56	100	67	53	100	65
Metacarpal base 3	5 (5)	1 (1)	2 (2)	20	100	81	20	94	76	25	94	80
Metacarpal base 4	8 (8)	3 (3)	2 (2)	38	100	76	25	100	71	20	100	78
Metacarpal base 5	7 (7)	2 (2)	2 (2)	29	100	76	14	93	67	20	93	74
Total	151 (166)	38 (43)	104 (119)	24	99	63	61	93	77	59	93	79

Erosion-like changes were registered in two healthy controls on CT, while one healthy control had three erosion-like changes on MRI (the same control also had erosion-like changes on CT) and none were seen on radiography.

Persons had a wide spectrum of joint destructions as judged on their erosions scores. The total OMERACT erosion score of one wrist (0–150) in all 21 persons was a mean of 10 (median 5, range 0–108) on MRI, while the mean was 15 (median 8, range 0–103) on CT. The total Sharp/van der Heijde erosion score (modified as mentioned in Materials and methods) produced a mean of 4 (median 1, range 0–43).

### Erosion volume

Results on erosion volume measurements and values on intramodality agreement of reading A and reading B (CT vs CT and MRI vs MRI) and intermodality (CT vs MRI) agreements are presented in Table 2. The intramodality agreements of single erosion volume measurements at the two occasions were very high for both CT (Spearman's ρ = 0.92, *P *< 0.01) and MRI (ρ = 0.90, *P *< 0.01). The intramodality agreements of persons' total erosion volume were also very high for CT (ρ = 0.83) and MRI (ρ = 0.80) (both *P *< 0.01). Volumes of erosions seen on both CT and MRI (concordant erosions) were compared. The volumes of the concordant erosions (*n *= 64) were correlated (ρ = 0.55, *P *< 0.01), as were the total volumes of concordant erosions on CT and MRI in the 15 persons with at least one concordant erosion (ρ = 0.89, *P *< 0.01). A significant correlation (ρ = 0.82, *P *< 0.01) between persons' (*n *= 19) total erosion volume on CT and MRI was also observed if all erosions – that is, not only concordant erosions – were included in the analysis.

**Table 2:** Intramodality and intermodality agreements of single and total erosion volume, measured on computed tomography (CT) and magnetic resonance imaging (MRI)

	Reading A (mm^3^)	Reading B (mm^3^)	Mean of readings A and B (mm^3^)	Spearman ρ	Absolute difference (mm^3^)^a^	Absolute numerical difference (mm^3^)	Relative difference (%)^b^	Relative numerical difference (%)	Coefficient of variation
**Intramodality agreement: CT (reading A) vs CT (reading B) and MRI (reading A) vs MRI (reading B)**									
Volume per erosion									
CT (*n *= 135)	13 (4; 1–245)	14 (4; 1–264)	13 (4; 1–255)	0.92*	-1 (0; -28 to 12)	2 (1; 0–28)	-7 (0; -120 to 100)	29 (22; 0–120)	0.15 (0.11; 0–0.60)
MRI (*n *= 90)	17 (10; 1–132)	17 (11; 1–138)	17 (11; 1–133)	0.90*	0 (0; -23 to 18)	4 (3; 0–23)	0 (0; -100 to 86)	28 (25; 0–100)	0.14 (0.13 0–0.50)
Volume per person with erosions									
CT (*n *= 17)	102 (49; 2–519)	108 (56; 3–535)	105 (55; 3–527)	0.99*	-6 (-2; -54 to 19)	12 (7; 1–54)	-10 (-6; -43 to 15)	16 (15; 3–43)	0.08 (0.07; 0.02–0.21)
MRI (*n *= 15)	101 (80; 5–409)	100 (78; 5–409)	100 (76; 5–409)	0.95*	1 (0; -23 to 18)	7 (5; 0–23)	2 (0; -22 to 25)	8 (6; 0–25)	0.04 (0.03; 0–0.13)
**Intermodality agreement (CT vs MRI)**^c^									
Volume per erosion of all concordant erosions (*n *= 64)									
CT	21 (5; 1–245)	22 (5; 1–255)	21 (5; 1–255)						
				0.55*	2 (-5; -55 to 132)	17 (9; 0–131)	-54 (-59; -174 to 167)	90 (92; 0–174)	0.46 (0.45; 0–0.87)
MRI	20 (13; 1–132)	19 (13; 1–138)	19 (13; 1–133)						
Total volume per person of all concordant erosions (*n *= 15)									
CT	88 (18; 1–514)	93 (23; 1–528)	91 (21; 1–521)						
				0.89*	8 (-7; -56 to 147)	40 (16; 4–147)	-48 (-63; -139 to 64)	72 (64; 6–139)	0.36 (0.32; 0.03–0.70)
MRI	83 (78; 5–374)	83 (62; 5–375)	83 (71; 5–375)						
Total volume per person of all erosions (*n *= 19)									
CT	91 (36; 0–519)	100 (49, 0–535)	94 (38; 0–527)						
				0.82*	15 (2; -60 to 118)	41 (31; 0–118)	13 (6; -129 to 200)	68 (52; 0–200)	0.34 (0.26; 0–1.0)
MRI	79 (67; 0–409)	79 (67; 0–409)	79 (63; 0–409)						

Data presented as the mean (median; range). Reading A and reading B, volumes obtained at the first (reading A) and second (reading B) volume measurements, done by the same observer 1 week apart. The mean value of volumes obtained at reading A and B was used for the comparison of CT and MRI volumes. *P < 0.01. ^a^Intramodality agreement, reading A minus reading B; intermodality agreement, CT erosion volume minus MRI erosion volume. ^b^Intramodality agreement, positive values refer to larger erosion volume at reading A than reading B, and *vice versa*; intermodality agreement, positive values refer to larger erosion volume on CT than MRI, and *vice versa*. ^c^Values on intermodality agreement are comparisons between CT and MRI erosions volumes.

### Erosion volume versus the OMERACT erosion score

The OMERACT erosion scores in the 15 evaluated wrist joint bones of the 19 examined persons (*n *= 285) were compared with the corresponding erosion volumes. The Spearman's correlation coefficients for CT and MRI erosion volumes and the corresponding OMERACT CT and MRI scores were 0.96 and 0.99 (both *P *< 0.01), respectively, when considering all 285 areas. When more than one erosion was present in a bone, the sum of the volumes of erosions in the bone was used for comparison with the OMERACT score. The total erosion volume per person (*n *= 19) and the total OMERACT erosion score of the wrist were closely correlated, as Spearman's correlation coefficients between volumes and scores on CT and MRI were 0.83 and 0.80, respectively (both *P *< 0.01). The correlation between the total MRI erosion score and the erosion volume determined on CT was ρ = 0.70 (*P *< 0.01).

### Erosion volumes and OMERACT erosion scores versus radiographic erosion scores

The correlation coefficients between the radiographic erosion score of the individual wrist bones (*n *= 285), according to the principles of the Sharp/van der Heijde scoring method, and the erosion volume in the corresponding bone, as measured on CT and MRI, were ρ = 0.27 (*P *< 0.01) and ρ = 0.10 (*P *= 0.10), respectively. Persons' total Sharp/van der Heijde erosion score of all wrist bones in all persons (*n *= 19) correlated with the total erosion volume on CT (ρ = 0.73, *P *< 0.01) and MRI (ρ = 0.70, *P *< 0.01).

The Sharp/van der Heijde erosion score of the individual wrist bones correlated weakly with the OMERACT erosion score on CT (ρ = 0.27, *P *< 0.01) but did not correlate with the MRI OMERACT erosion score (ρ = 0.10, *P *= 0.11). The persons' total Sharp/van der Heijde erosion score, however, correlated with the total OMERACT erosion score on both CT (ρ = 0.83, *P *< 0.01) and MRI (ρ = 0.66, *P *< 0.01).

## Discussion

With CT as the standard reference method for detecting bone erosions in wrist joints, a moderate sensitivity (61%) and a high specificity (93%) of MRI was demonstrated. Although radiography was also highly specific (99%), only a low sensitivity (24%) for erosions was reached when compared with CT. The very low sensitivity of radiography, as compared with CT, found in the present study can be explained by the two-dimensional visualisation of the joint, and is in accordance with findings from previous comparisons with MRI [9-13,23] and with CT [12,23].

Since the amount of mobile protons in bone is very low, cortical bone is depicted on MRI as signal voids against signal-emitting bone marrow and periosseous tissues. MRI has consequently been argued not to be a method well suited for visualising bone lesions, and the nature of erosions visualised with MRI but invisible on radiography has been questioned [22]. In the present study, however, MRI was markedly more sensitive than radiography and was in good agreement with CT even in regions without radiographic erosions, supporting that even radiographically invisible MRI erosions represent a true loss of calcified tissue.

The even higher agreement (87% vs 77%) between CT and MRI in a study of nine RA wrist joints by Perry and colleagues [12] may partly be explained by more advanced joint destructions in their cohort. In comparison with our previous study on RA metacarpophalangeal joints [23], the level of agreement between CT and MRI in the present study was lower. The anatomy of the wrist is much more complicated than that of the metacarpophalangeal joints, and many of the small carpal bones have irregular margins with indentations (for example, at the attachment of ligaments), making discrimination between normal anatomy and presence of erosions difficult, and nutritive foramina may also resemble erosions [28]. This may, at least partly, explain the lower sensitivity and accuracy in this wrist joint study compared with previous results from metacarpophalangeal joints [23]. In the present study, erosion-like changes were registered in two healthy controls on CT and in one healthy control on MRI. A low prevalence of erosion-like changes on MRI in healthy controls has previously been reported for wrists and metacarpophalangeal joints [29]. A 0.6 T (midfield) MRI unit was used in the present study. We expect values on sensitivities and specificities on erosions are also applicable to MRI units using higher field strengths, since previous studies have showed comparable results when images obtained on MRI units with higher field strengths were compared with images obtained on low-field MRI units [30-32].

As the wrist joint has proved more sensitive to changes in bone erosions than other joint areas in RA [33], and bone changes in the wrist joint have been shown to possess predictive value with respect to further radiographic erosive progression [14,34,35], we found the wrist an important joint area to investigate.

The number of erosions detected on CT, as compared with MRI and radiography, indicate that CT is a very sensitive method for detecting bone erosions in RA wrist bones, and possibly even more sensitive than MRI. CT may therefore be of value for detecting and monitoring bone erosions in RA. The sensitivity to change is not yet established, however, and CT is disfavored by using ionising radiation and by the inability to visualise soft tissue changes.

**Figure 1:**
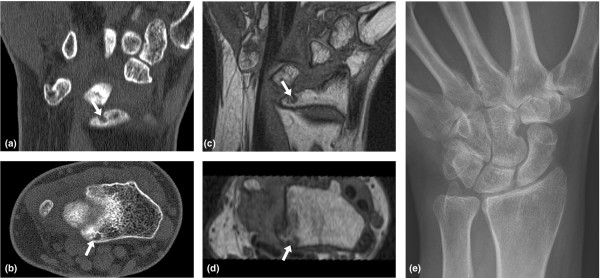
Erosions in the wrist of a rheumatoid arthritis patient. Wrist of a rheumatoid arthritis patient visualised by **(a, b) **computed tomography and **(c, d) **T1-weighted magnetic resonance imaging in the **(a, c) **coronal and **(b, d) **axial planes. A bone erosion at the distal radius is seen on both computed tomography and magnetic resonance images in two planes (white arrows), but not on the corresponding radiograph **(e)**. The erosion was assigned an OMERACT erosion score of 1 on both computed tomography and magnetic resonance imaging.

We recently published data on the reliability of erosion volume measurements on CT and MRI in RA metacarpophalangeal joints, showing very high reproducibility when measuring erosion volumes on CT and MRI, and good correlations between CT and MRIerosion volumes and between erosion volumes and erosion scores. [21] The present study on RA wrist joints also showed a very high level of reproducibility when measuring volumes of erosions on CT and MRI. Furthermore, semiquantitative scores of bone erosions according to the OMERACT scoring system were closely correlated with both CT and MRI volumes, both for individual joint regions and for the wrist joint as a whole, supporting that the OMERACT erosion score reflects the extent of erosive joint damage.

Although high to very high agreements of volumes were reached between and within imaging modalities (respectively), there were individual measurements that differed markedly. Coincidental differences in outlining erosions at the two time points are potentially a major source of error. Especially, the peripheral border of erosions can be difficult to define as signal intensities of erosions and adjacent soft-tissues often are very similar for both CT and magnetic resonance images. Generally, the larger and more advanced the erosion, the more difficult it was to define the exact border of the erosions. The estimated erosion volumes of concordant erosions were, on average, larger on MRI than CT, as reflected by the mean relative difference in erosions' size. As cortical bone appears black on MRI it may be included in the outlining of erosions, and may consequently lead to overestimation of erosion size on MRI compared with CT, where the cortical bone is well delineated. Furthermore, the majority of erosions in the present study were small; for small erosions, small absolute differences will result in large relative differences, with a systematic bias towards larger volumes on MRI due to a proportionally large area of cortical bone included in the estimation of erosion size. The total erosion volume, however, was relatively larger on CT than MRI due to more erosions being detected with CT.

Using the OMERACT RAMRIS, Haavardsholm and colleagues have recently shown very good intrareader and good interreader reliability, and a high level of sensitivity to change- demonstrating that the OMERACT RAMRIS system, after proper training and calibration of readers, appears suitable for use in monitoring joint inflammation and destruction in RA [36]. The close correlation with erosion volumes determined by MRI, as well as CT, provides further important evidence of the OMERACT RAMRIS erosion score being a valid measure of RA bone destruction.

## Conclusion

The present study demonstrated a high specificity of bone erosions detected on MRI and radiography, and showed a markedly higher sensitivity of MRI than radiography when CT was considered the reference method. Secondly, when measuring erosion volumes by CT and MRI, a very high intramodality and a high intermodality agreement was reached, applying both to individual erosion volume and persons' total erosion volume. Owing to the high reproducibility, this quantitative method for assessing bone erosions in RA patients could be a useful tool in longitudinal studies, including randomised controlled trials, but further studies, including studies of sensitivity to change, are needed to clarify this issue. As the OMERACT erosion scores were closely correlated with erosion volumes determined on CT and MRI, the present study supports the OMERACT erosion score as a valid measure of RA joint destruction.

## Abbreviations

CT = computed tomography; MRI = magnetic resonance imaging; OMERACT = Outcome Measures in Rheumatology; RA = rheumatoid arthritis; RAMRIS = Rheumatoid Arthritis MRI Scoring System.

## Competing interests

The authors declare that they have no competing interests.

## Authors' contributions

UMD participated in the study development and recruitment of patients, performed erosion volume measurements, conducted data evaluation and statistical analysis, and prepared the manuscript draft. BJE participated in the study development, performed the evaluation of magnetic resonance images, and was involved in patient recruitment. MH was involved in the CT scanning protocol. EN performed the evaluation of radiographs. JM was involved in the MRI scanning protocol and performed all MRI examinations. HST participated in the study development and gave substantial input to data evaluation and manuscript preparation. MØ participated in the study development, was involved in the CT and MRI scanning protocol, evaluated CT images, and gave substantial input to data evaluation and manuscript preparation. All authors read and approved the final manuscript.
